# When eyes beat lips: speaker gaze affects audiovisual integration in the McGurk illusion

**DOI:** 10.1007/s00426-021-01618-y

**Published:** 2021-12-02

**Authors:** Basil Wahn, Laura Schmitz, Alan Kingstone, Anne Böckler-Raettig

**Affiliations:** 1 Department of Psychology, Leibniz Universität Hannover, Hannover, Germany; 2 Institute of Sports Science, Leibniz Universität Hannover, Hannover, Germany; 3 Department of Psychology, University of British Columbia, Vancouver, BC Canada

## Abstract

Eye contact is a dynamic social signal that captures attention and plays a critical role in human communication. In particular, direct gaze often accompanies communicative acts in an ostensive function: a speaker directs her gaze towards the addressee to highlight the fact that this message is being intentionally communicated to her. The addressee, in turn, integrates the speaker’s auditory and visual speech signals (i.e., her vocal sounds and lip movements) into a unitary percept. It is an open question whether the speaker’s gaze affects how the addressee integrates the speaker’s multisensory speech signals. We investigated this question using the classic McGurk illusion, an illusory percept created by presenting mismatching auditory (vocal sounds) and visual information (speaker’s lip movements). Specifically, we manipulated whether the speaker (a) moved his eyelids up/down (i.e., open/closed his eyes) prior to speaking or did not show any eye motion, and (b) spoke with open or closed eyes. When the speaker’s eyes moved (i.e., opened or closed) before an utterance, and when the speaker spoke with closed eyes, the McGurk illusion was weakened (i.e., addressees reported significantly fewer illusory percepts). In line with previous research, this suggests that motion (opening or closing), as well as the closed state of the speaker’s eyes, captured addressees’ attention, thereby reducing the influence of the speaker’s lip movements on the addressees’ audiovisual integration process. Our findings reaffirm the power of speaker gaze to guide attention, showing that its dynamics can modulate low-level processes such as the integration of multisensory speech signals.

## Introduction

### The role of gaze in social interaction

The eyes of another person are of fundamental importance to human behavior from early infancy on (Farroni et al., 2004; Striano & Reid, 2006), shaping attentional, perceptual, and affective processing. If someone directs their gaze towards us, our attention is immediately captured. Direct gaze (as opposed to averted gaze or closed eyes) is preferentially detected and processed (e.g., Baron-Cohen, 1995; Coelho et al., 2006; Senju & Hasegawa, 2005; von Grünau & Anston, 1995; also see Böckler et al., 2015). Being looked at by another person may elicit self-referential processing in the addressee (Conty et al., 2016; Hietanen & Hietanen, 2017) and boost prosocial behavior (Izuma et al., 2010, 2011) as well as positive appraisal of others (see Kleinke, 1986, for a review). Another’s eyes convey information about their affective and cognitive states (e.g., Emery, 2000; Kleinke, 1986; Schilbach, 2015) and can signal aggression (Nichols & Champness, 1971) as well as attraction (Mason et al., 2005). Critically, the eyes not only *signal* information to others but they simultaneously *encode* information from the environment, thus serving a dual function (Cañigueral & Hamilton, 2019; Gobel et al., 2015; Kendon, 1967; Risko et al., 2016).

Not surprisingly, eye contact plays a major role in human communication. In particular, direct gaze serves as an ostensive communicative signal (Csibra & Gergely, 2009; Sperber & Wilson, 1986): A speaker typically directs her gaze towards the addressee, thereby making it manifest to the addressee that she is the designated recipient of the upcoming message and that this message is being intentionally communicated to her (Csibra & Gergely, 2009; Lanthier et al., 2019, 2021). Moreover, the gaze is used in a conversation to control turn-taking behavior such that a speaker ends her turn with a direct gaze at the addressee and the addressee then begins her turn with averted gaze (Kendon, 1967; recently replicated by Ho et al., 2015). When responding to questions, preferred responses are produced while gazing at the questioner whereas the gaze is averted for dispreferred responses (Kendrick & Holler, 2017). To signal understanding in conversation, addressees systematically use eye blinking (Hömke et al., 2017). It has further been found that the stronger the coupling between speakers’ and addressee’s eye movements, the better the addressee’s comprehension (Richardson & Dale, 2005).

### Multisensory integration in speech perception

While the processing of a speaker’s *gaze* is important in a conversation, the processing of the speaker’s *speech* is naturally paramount. During speech perception, addressees integrate the incoming auditory and visual signals (i.e., the speaker’s vocal sounds and lip movements) into a unitary percept through a process known as *multisensory integration* (Ernst & Bülthoff, 2004). Generally, whether our brain binds multiple incoming sensory signals together or processes them separately depends on whether these signals are perceived to be causally related. When we hear another person’s words while seeing their lips move, the visual signal (the lip movements) and the auditory signal (the vocal sounds) have the same causal origin (the other person) and are integrated into a single percept rather than perceived as two separate signals. Typically, the integration of signals from multiple sensory modalities boosts perceptual performance because several sources of redundant information are combined. For instance, speech in a noisy environment is understood better if the speaker’s lip movements can be observed by the addressee (Ma et al., 2009; MacLeod & Summerfield, 1987; Ross et al., 2007; also see Altieri et al., 2016).

If, however, the signals from multiple sensory modalities provide inconsistent information, the process of multisensory integration can impair or bias perceptual performance and can lead to sensory illusions (e.g., Shams et al., 2000). In the case of speech perception, if the visual and auditory signals do not match, this can give rise to auditory percepts that neither correspond to the visual nor the auditory signal. In particular, McGurk and MacDonald (1976) have shown that when participants were presented with an auditory syllable (e.g. “Ba”) while observing the speaker’s lips uttering a different syllable (e.g. “Ga”), they reported hearing an illusory syllable (e.g. “Da”). This striking multisensory illusion (often referred to as the “McGurk illusion” or “McGurk effect”) demonstrated, for the first time, the powerful influence of vision upon auditory speech perception. The McGurk illusion has since been extensively investigated with regard to the mechanisms underlying it and the factors influencing it (for a comprehensive review, see Alsius et al., 2018). Note that the illusion has also been reliably found in studies that were run online (e.g., Brown et al., 2018; Karas et al., 2019; Magnotti et al., 2018, 2020), with highly similar results between lab-based and online studies (Magnotti et al., 2018). Previous research suggests that the McGurk illusion can be explained using a causal inference model of multisensory perception (Magnotti & Beauchamp, 2017) that has also been applied successfully to a variety of other multisensory phenomena, such as the ventriloquist effect (Körding et al., 2007; Rohe & Noppeney, 2015) and the sound-induced flash illusion (Shams et al., 2005).

In terms of factors that influence the McGurk illusion, previous studies have shown that attentional demands play a critical role. In particular, if participants were presented with the typical McGurk stimuli and asked to indicate what they heard while at the same time performing a secondary (visual or auditory) task, the perception of the McGurk illusion decreased (Alsius et al., 2005). This finding indicates that audiovisual integration of speech is weakened under a high attentional load. Relatedly, another study (Munhall et al., 2009) showed that the McGurk illusion is perceived only if addressees *consciously attend* to the visual signal, i.e., the speaker’s lip movements. Together, these findings suggest that one needs to pay (a sufficient amount of) attention to the presented stimuli in order for the integration process to take place.

Whereas dual tasks like the above (Alsius et al., 2005) explicitly shift and divide participants’ attentional resources, a recent study by Gurler et al. (2015) investigated the natural (i.e., uninfluenced) distribution of people’s visual attention while they observed the face of the “McGurk speaker” uttering syllables. Via eye tracking, the authors measured which region on the speaker’s face participants tended to fixate on, using eye fixation as a proxy for attentional focus. The results showed that the distribution of participants’ eye fixations predicted the degree to which participants perceived the McGurk illusion. Specifically, the more participants tended to look at the speaker’s mouth, the more they perceived the McGurk illusion—presumably because they were more strongly influenced by the visual signal provided by the lip movements (for a replication and additional manipulations, see Stacey et al., 2020). Hence, observing the speaker’s lip movements—a strategy that would usually improve speech comprehension (Ma et al., 2009; MacLeod, & Summerfield, 1987; Ross et al., 2007)—has a negative and misleading effect because the visual signal coming from the lips is inconsistent with the auditory signal.

Interestingly, Gurler et al. (2015) also observed that there were several participants who did not look primarily at the speaker’s mouth, choosing instead to look at the speaker’s eyes or explore multiple regions of the face*.* These participants perceived the McGurk illusion to a lesser extent. Thus, this study suggests that there is no commonly shared focus of attention in the McGurk paradigm, i.e., participants tend to look at the mouth, or at the eyes, or at multiple face regions. Hence, one cannot predict in advance where a particular participant will focus her attention when confronted with the McGurk stimulus.

In sum, previous research by Alsius et al. (2005), Gurler et al. (2015), Munhall et al. (2009), and Stacey et al. (2020) suggests that the degree to which people perceive the McGurk illusion depends (1) on their attention in general (with less attention leading to a decrease of the illusion) and (2) on their attentional focus on the speaker’s mouth versus elsewhere (with a focus on the mouth leading to an increase of the illusion).

### Does speaker gaze affect multisensory integration in speech perception?

The eyes of another person have—amongst other things—the function and the power to spontaneously direct, divert, or capture our attention. In particular, another’s direct eye gaze and motion onset (e.g., switching from direct to averted gaze, or from closed to open eyes) are two powerful cues that capture our attention (e.g., Abrams & Christ, 2003; Böckler et al., 2014; Senju & Hasegawa, 2005). In turn, what we attend (and to what extent we attend it) determines how we process incoming multisensory signals (e.g., Alsius et al., 2005; Munhall et al., 2009) – and thus affects whether we can be “tricked” by our senses and subjected to multisensory illusions (Gurler et al., 2015; Stacey et al., 2020).

During a conversation, a speaker’s eyes and the audiovisual signals coming from their lips both typically provide relevant information. The addressee processes the speaker’s gaze while at the same time processing her audiovisual speech signals. To date, it has not been systematically investigated whether these two processes interact, i.e., whether the speaker’s gaze affects how the addressee integrates the speaker’s audiovisual speech signals. In the present study, we aimed to address this question using the classic McGurk illusion. Building on previous research, we examined if a speaker’s gaze behavior (i.e., motion and eye contact) would dynamically capture an addressee’s attention and would thereby influence how the addressee integrates the speaker’s vocal sounds and lip movements.

To test the effect of motion, we manipulated whether the speaker moved his eyelids up/down (i.e., open/closed his eyes) prior to speaking or did not show any eye motion (factor “Motion”). To test the effect of eye contact, we manipulated whether the speaker spoke with open eyes or closed eyes (factor “Eyes”). The factor Motion was manipulated between-subjects, the factor Eyes was manipulated within-subjects.

#### Predictions for motion

If the sudden onset of motion in the eye region draws the addressee's attention to the speaker’s eyes (and away from the lips), we expect a reduced McGurk illusion when the speaker opens or closes his eyes prior to speaking compared to when there is no motion of the eyes. The misleading visual signal from the lips receives less attention in this case and thus influences the audiovisual integration process to a lesser extent, resulting in a more accurate perception of the auditory syllable (cf. Gurler et al., 2015; Munhall et al., 2009).

#### Predictions for eyes

Similarly, if a speaker’s direct gaze draws the addressee’s attention to the speaker’s eyes, as shown by previous research (e.g., Böckler et al., 2014, who used face pictures), we expect a *reduced* McGurk illusion when the speaker has open eyes as compared to closed eyes.

Alternatively, however, one could predict that a speaker’s direct, ostensive gaze leads to a *general* increase in the addressee’s attention because she feels personally addressed and is eager to understand the speaker’s message (cf. Csibra & Gergely, 2009; Lanthier et al., 2019, 2021). When the speaker speaks with closed eyes, the addressee might not consider herself the intended recipient of the message and thus not pay as much attention. Given that attention is essential for audiovisual integration to occur in the first place (Alsius et al., 2005; Munhall et al., 2009; Talsma et al., 2007) and that selective attention enhances the integration process (Talsma & Woldorff, 2005), this pattern of behavior would result in the addressee experiencing an *enhanced* McGurk illusion when the speaker has open eyes (addressee pays close attention) as compared to closed eyes (addressee pays reduced attention).

Two further considerations support the latter prediction of enhanced McGurk illusion when the speaker has open eyes, yet for different reasons. First, being looked at by another person increases self-referential processing (Conty et al., 2016; Hietanen & Hietanen, 2017), self-awareness (e.g., Baltazar et al., 2014; Hazem et al., 2017; Pönkänen et al., 2011), and arousal (Helminen et al., 2011; Hietanen et al., 2020). Accordingly, looking into a speaker’s open eyes is more demanding for the addressee than looking at a speaker’s closed eyes. Second, it is possible that a speaker’s closed eyes capture the addressee’s attention because closed eyes in a conversation are very unusual for a speaker and thus salient for an addressee. Together, these two points suggest that an addressee might focus more on a speaker’s eyes (and thus less on his lips) when they are closed as opposed to open. In this case, the addressee would be less susceptible to the McGurk illusion when the speaker’s eyes are closed. Thus, one should expect an *enhanced* McGurk illusion when the speaker has open eyes as compared to closed eyes.

In sum, the theoretical considerations spelled out above provide grounds for a bidirectional prediction for the factor Eyes. On the one hand, one can predict that a speaker’s open eyes will draw the addressee’s attention (away from the speaker’s lips), leading to a *smaller McGurk illusion for open compared to closed eyes*. On the other hand, one can predict that (1) a speaker’s open eyes will generally lead to higher levels of attention in the addressee and (2) a speaker’s closed eyes will draw the addressee’s attention (away from the speaker’s lips). Both (1) and (2) would lead to a *larger McGurk illusion for open compared to closed eyes*.

## Methods

### Participants

We determined our target sample size of 2 × 70 participants by running an a priori power analysis using G*Power (Faul et al., 2007, 2009) targeting moderately sized effects (Cohen’s *d* = 0.34 for paired samples *t*-tests; Cohen’s *d* = 0.48 for independent samples *t*-tests; alpha = 0.05, Power = 0.80). Thus, we recruited 70 participants for each level of our between-subjects factor Motion, i.e., 70 for the “Static” condition (motion absent) and 70 for the “Dynamic” condition (motion present). Data was collected through the online participant recruitment service *Prolific* (https://www.prolific.co/).^1^ All participants had normal or corrected-to-normal vision and hearing; they were between 18 and 45 years old and fluent in English.^2^ Only participants whose performance in previous *Prolific* studies had been reliable (approval rates of at least 75%) were admitted to this study. Moreover, participants who showed below 60% accuracy in an auditory baseline condition of our study were excluded because we needed to ensure intact hearing capabilities.

The participant samples for the Static and the Dynamic conditions consisted of 33 females, 36 males, and 1 other (*M* = 25.47 years, *SD* = 6.16 years), and of 21 females, 48 males, and 1 other (*M* = 24.30 years, *SD* = 6.33 years), respectively. All participants gave written informed consent and received monetary compensation for their participation (1.50 GBP for the Static condition (~ 12 min) and 1.88 GBP for the slightly longer Dynamic condition (~ 15 min)).

### Apparatus and stimuli

**Fig. 1:**
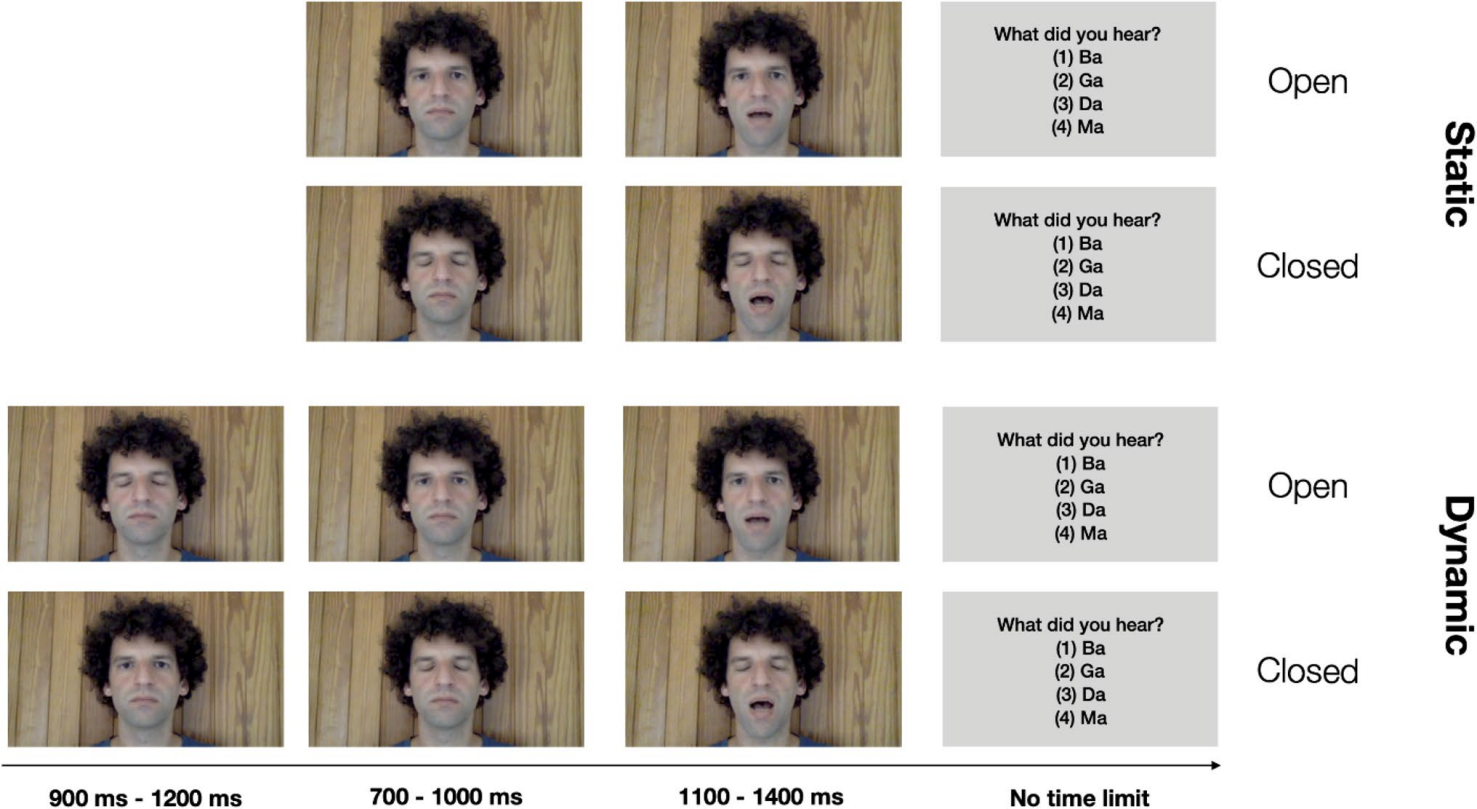
Example trial sequence for each level of the factors Motion (Static/Dynamic) and Eyes (Open/Closed)

Videos were recorded with a *MacBook* (early 2016) using its internal microphone and its 480p *FaceTime* camera. Each video showed a headshot of a man (see Fig. 1) uttering one out of five syllables (“Ba”, “Pa”, “Ga”, “Ka”, “Na”). The speaker’s eyes were either open (“Eyes open”) or closed (“Eyes closed”) while he was speaking. In the Dynamic condition, the speech act was preceded by motion: the speaker either opened or closed his eyes before uttering the syllable (with his eyes remaining open or closed). Each video clip lasted 3.5 s. To create a second version of the videos that did not contain the preceding motion (Static condition), the respective first part of each clip was cut off, resulting in a shorter (2 s) version of each clip. Thus, the long video clips showed the speaker first closing or opening his eyes and then uttering one of the syllables with either open or closed eyes (Dynamic) whereas the shorter clips only included the speech act without the preceding motion (Static), see Fig. 1.

These video clips naturally included matching auditory and visual signals (e.g., the speaker’s lips produced the word “Ga” and the auditory signal was “Ga”). In addition to these “congruent” versions, we created “incongruent” versions with mismatching auditory and visual signals. To this end, the actual sounds in the videos were muted and dubbed either with the auditory syllable “Ba” or “Pa” (using *iMovie*, version 10.1.9) such that, for example, the speaker’s lips produced the word “Ga” yet the auditory signal was “Ba” or “Pa”. The final set of audiovisual stimuli included 6 incongruent versions and 6 congruent versions (see Table 1 for an overview). Additionally, we used only the audio tracks for the syllables “Ba” and “Pa” as an auditory baseline (see “auditory only” in Table 1).

**Table 1:** Syllable combinations and AFC response options (adopted from Stropahl et al., 2017)

Auditory–visual	4-AFC options (auditory, visual, Fusion1, Fusion2)	Trial type
Ba–Ga	Ba, Ga, Da, Ma	Incongruent
Ba–Ka	Ba, Ka, Ga, Da	Incongruent
Ba–Na	Ba, Na, Ga, Da	Incongruent
Pa–Ga	Pa, Da, Ka, Ta	Incongruent
Pa–Ka	Pa, Ka, Da, Ta	Incongruent
Pa–Na	Pa, Na, Ka, Ta	Incongruent
Ba–Ba	Ba, Ga, Da, Ma	Congruent
Ba–Ba	Ba, Ka, Ga, Da	Congruent
Ba–Ba	Ba, Na, Ga, Da	Congruent
Pa–Pa	Pa, Da, Ka, Ta	Congruent
Pa–Pa	Pa, Ka, Da, Ta	Congruent
Pa–Pa	Pa, Na, Ka, Ta	Congruent
Ba–None	Ba, Ga, Da, Ma	Auditory only
Ba–None	Ba, Ka, Ga, Da	Auditory only
Ba–None	Ba, Na, Ga, Da	Auditory only
Pa–None	Pa, Da, Ka, Ta	Auditory only
Pa–None	Pa, Ka, Da, Ta	Auditory only
Pa–None	Pa, Na, Ka, Ta	Auditory only

All video material was recorded in one continuous take to ensure similar intonation and loudness of the uttered syllables and to keep visual conditions constant. The speaker opened and closed his eyes and produced the syllables in sync with a metronome beat (40 bpm; not audible to participants) to ensure that the temporal sequences were comparable across videos. This way, we also attempted to exclude potential tempo differences between speaking with open vs. with closed eyes. All videos are publicly accessible (under CC-BY license) via the Open Science Framework (https://osf.io/vjw6k/?view_only=7c4c9831aa6a4dbb8fcfbd5a8ef7e501).

The experiment was programmed in *PsychoPy 3* (Peirce et al., 2019) and run online via *Pavlovia (*https://pavlovia.org/*)*.

### Design and procedure

To test whether motion and eye contact affect multisensory speech processing, we used a 2 × 2 mixed factorial design. As a between-subjects factor, we varied whether motion (i.e., opening or closing of the speaker’s eyes) preceded the speech act or not (Motion: Dynamic/Static). As a within-subjects factor, we varied whether the speaker’s eyes were open or closed while he was uttering the syllable (Eyes: Open/Closed).

Participants were presented with three different trial types (incongruent, congruent, auditory only) in randomized order, see Table 1. In the incongruent trials, the syllable that the speaker produced with his lips (either “Ga”, “Ka”, or “Na”) did not match the auditory syllable that was presented (“Pa” or “Ba”). In the congruent trials, the syllable that the speaker produced matched the auditory syllable (“Pa” or “Ba”). In the auditory only trials, participants were presented with a black screen (instead of the speaker’s face) and either heard the syllable “Pa” or “Ba”. Note that we did not include “visual only” trials.

After stimulus presentation, participants were asked to indicate which syllable—out of four options presented on the screen—they heard (see Table 1, for a list of all syllable combinations and response options). The four response options were adopted from Stropahl et al. (2017) and comprised the presented auditory syllable, the presented visual syllable, and two fusion responses. The two fusion responses were the two responses that showed the highest fusion percentage (i.e., the most commonly reported illusory percepts resulting from the fusion of mismatching auditory and visual signals) in the original study by McGurk and MacDonald (1976). The response options were the same for all three trial types (see Table 1).

Participants responded by pressing the corresponding number of the response option (1, 2, 3 or 4) on the keyboard. There was no time limit for responses. Participants were told that if they were unsure about what the speaker said, they should simply choose the response option that seemed most likely to them. They were ensured that this task was not about accuracy but about their individual perception.

Participants performed a total of 120 trials. The order of response options for each trial type was randomly chosen out of two possible orders (either “Visual, Auditory, Fusion1, Fusion2” or “Fusion1, Fusion2, Visual, Auditory”). The options were presented in rows, one word beneath the other, and numbered consecutively from 1 to 4. The 120 trials were composed of 24 auditory only, 24 congruent, and 72 incongruent trials. Critically, in half of the congruent and in half of the incongruent trials, the speaker’s eyes were open (first and third row, Fig. 1); in the other half they were closed (second and fourth row, Fig. 1).

The only difference between the two experimental conditions was that in the Dynamic condition, the speaker either opened or closed his eyes prior to uttering the syllable (third and fourth row, Fig. 1). In the Static condition, there was no motion preceding the utterance (first and second row, Fig. 1). Exemplary trial sequences for all four-factor combinations (Static + Eyes open, Static + Eyes closed, Dynamic + Eyes open, Dynamic + Eyes closed) are shown in Fig. 1.

As part of the general study instructions, participants were asked to turn off all distractions (e.g., music, TV, phone) and to always *look* at the screen and *listen* to the voice. They were asked to concentrate fully on the task and not to perform any other tasks at the same time. The instructions pointed out that they should read the response options carefully because the available options and the order of options would change continuously. Finally, participants were asked to wear headphones, if possible, while completing the study.

Before starting the actual experiment, participants were familiarized with the trial logic by performing three training trials. The first two training trials were congruent trials and the third trial was an auditory-only trial.

As dependent variables, we recorded *response accuracies* (for congruent and auditory-only trials), *response choices* (for incongruent trials), and *response times*^3^ for all trials. Response accuracy was computed as the proportion of trials in which participants chose the auditory syllable (i.e., the vocal sound that is actually presented) out of the four response options. Response choice for incongruent trials (where auditory and visual signals mismatched) was computed as the proportion of trials in which participants chose the auditory syllable, the visual syllable (i.e., the sound originally produced by the lips), and the fused syllable (i.e., the illusory percept), respectively. Response time was computed as the time between stimulus offset (i.e., end of video and appearance of the response options) and response selection.

### Data analysis

For statistical inference, we used permutation-based ANOVAs and post-hoc tests. That is, the null distribution of the test statistics was estimated by repeatedly sampling permutations of the actual data under the assumption that there are no differences between the levels of our experimental factors (Kherad-Pajouh & Renaud, 2015). All post-hoc tests were Bonferroni-corrected. As effect size measures, we report generalized eta squared (η_G_^2^; Bakeman, 2005) for the ANOVAs and Cohen’s *d* for the post-hoc tests. Data were analyzed using customized *R* scripts.

All raw data are publicly available via the Open Science Framework (https://osf.io/vjw6k/?view_only=7c4c9831aa6a4dbb8fcfbd5a8ef7e501).

## Results

### Auditory baseline and congruent trials

#### Response accuracy

**Fig. 2:**
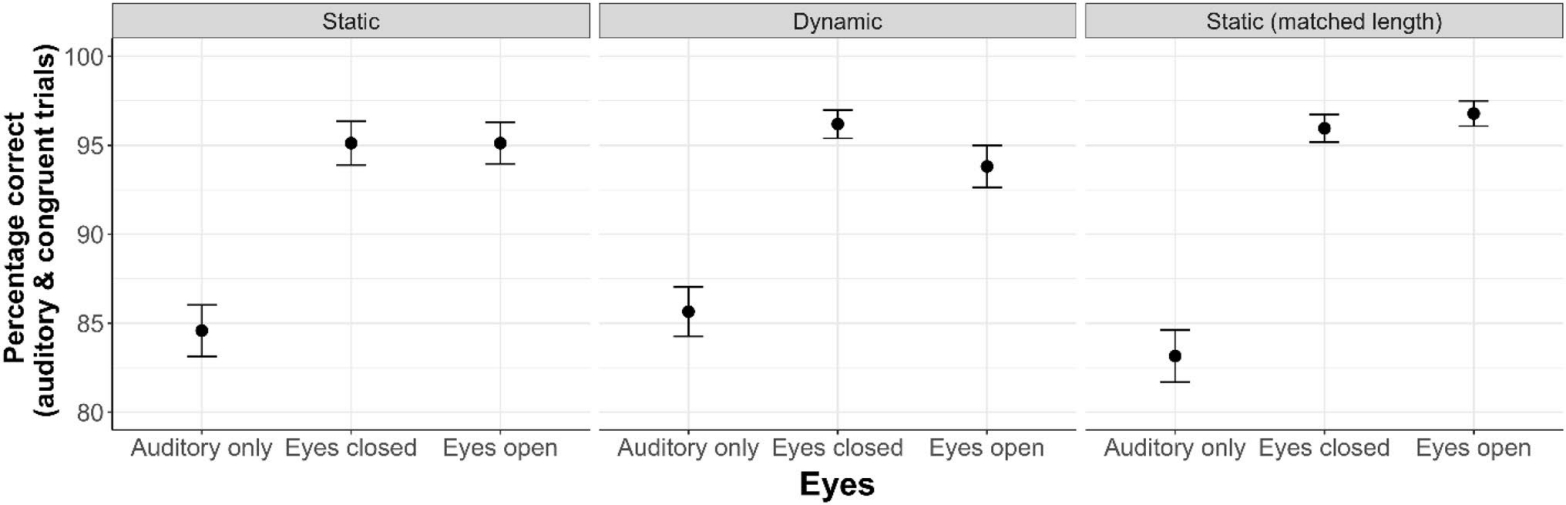
Accuracy (averaged percentage correct) in auditory and congruent trials is shown as a function of Eyes (Auditory only, Eyes closed, Eyes open) and Motion (Static, Dynamic, Static (matched length)). Error bars show the standard error of the mean. The Static condition with matched length, which was run as an additional control condition, is discussed in Sect. Control condition: Static matched

First, we aimed to verify that participants demonstrated the expected highly accurate performance in auditory-only trials and in congruent trials. In these trials, the identification of the presented syllable should be straightforward as there is no mismatching information. We found that participants in both conditions were highly accurate (*M* = 85%) in auditory-only trials and almost reached ceiling performance (*M* = 95%) in congruent trials (see Fig. 2).

To test whether accuracy levels differed statistically in the auditory-only trials and congruent trials, we performed an analysis including the factor Motion (Static/Dynamic) as between-subjects factor and the factor Eyes (Auditory only, Eyes open (congruent), and Eyes closed (congruent)) as within-subjects factor. This resulted in a 2 (Motion: Static/Dynamic) × 3 (Eyes: Auditory only, Eyes closed, Eyes open) ANOVA. The results showed a significant main effect of Eyes (*F*(2,276) = 81.65, *p* < 0.001, η_G_^2^ = 0.178) but no other significant effects (Motion: *F*(1,138) = 0.04, *p* = 0.840, η_G_^2^ < 0.001; Eyes x Motion: *F*(2,276) = 1.16, *p* = 0.315, η_G_^2^ = 0.003). We followed up the significant main effect of Eyes by performing pairwise comparisons between the three-factor levels using paired *t*-tests, separately for the Static and Dynamic condition. We found that participants showed a significantly lower accuracy in the auditory-only trials compared to the other two trial types in both the Static and the Dynamic condition (all *corrected p*s < 0.007; averaged Cohen’s *d* = 0.92); the other comparisons were not significant (all *corrected p*s > 0.199; averaged Cohen’s *d* = 0.14). In sum, these results show that participants could reliably identify the spoken syllables (“Ba” and “Pa”) in the auditory-only trials and that their performance was further boosted—as expected based on previous research (Ma et al., 2009; MacLeod, & Summerfield, 1987; Ross et al., 2007)—when compatible visual information (the speaker’s lip movements) was provided in the congruent trials. This performance boost occurred irrespective of whether the speaker’s eyes were closed or open and irrespective of whether prior motion occurred or not.

#### Response times

As a second step, we determined whether the differences in participants’ accuracy levels were reflected in their response times. Repeating the same 2 × 3 ANOVA as reported above with response times as dependent variable, we found no significant effects (Eyes: *F*(2,276) = 2.88, *p* = 0.058, η_G_^2^ = 0.002; Motion: *F*(1,138) = 0.08, *p* = 0.775, η_G_^2^ < 0.001; Eyes × Motion: *F*(2,276) = 0.29, *p* = 0.747, η_G_^2^ < 0.001). These results indicate that findings in accuracies were not due to a speed-accuracy trade-off.

### Incongruent trials

#### Response choices

**Fig. 3:**
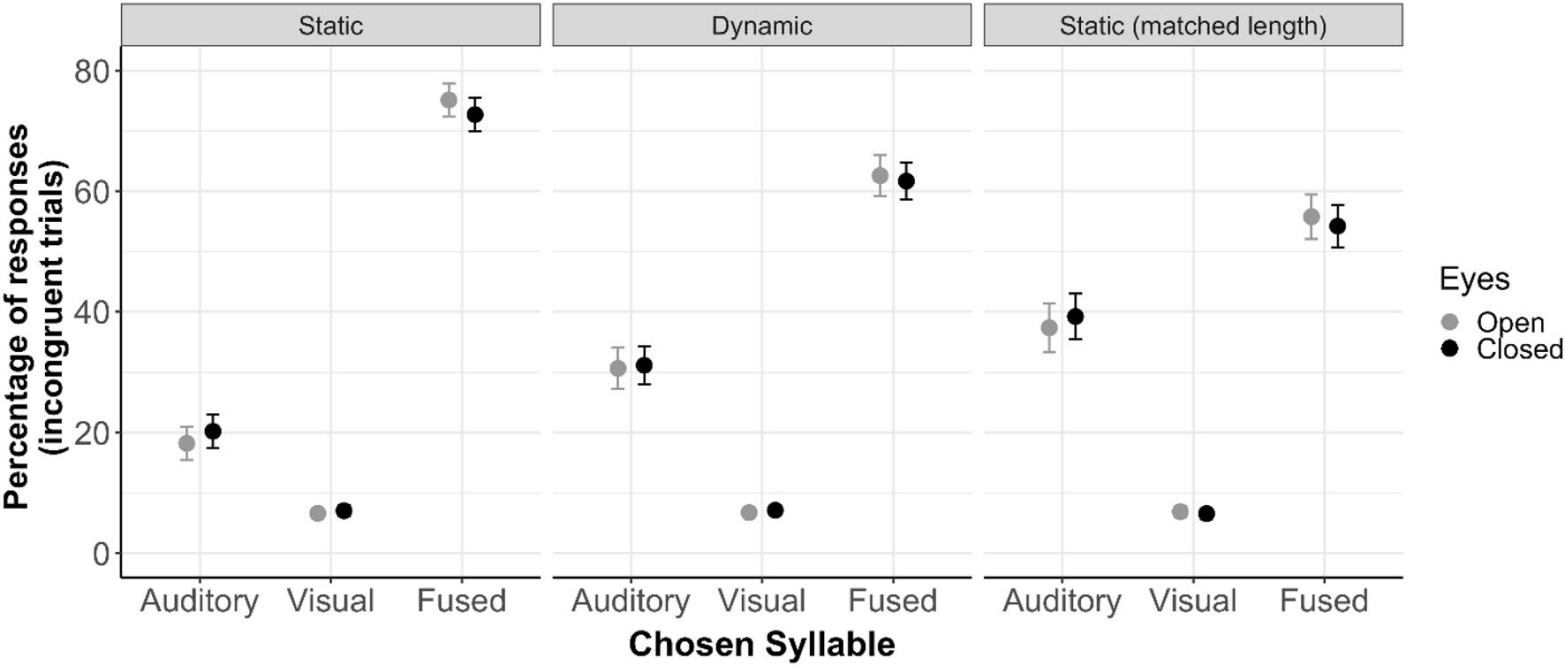
Percentage of total responses in incongruent trials is shown separately for Eyes open (grey) and Eyes closed (black), as a function of Chosen Syllable (Auditory, Visual, Fused (= illusory percept)) and Motion (Static, Dynamic, Static (matched length)). Error bars show the standard error of the mean. The Static condition with matched length, which was run as an additional control condition, is discussed in Sect. Control condition: Static matched

To test our main research question of whether motion and eye contact affect multisensory speech processing, we analyzed participants’ responses in the incongruent trials where auditory and visual signals mismatched. On a descriptive level, we observed that participants chose the fusion response in a high proportion of trials in both conditions (*M* = 68%), indicating that they did indeed experience the McGurk illusion, as expected based on previous research (McGurk & MacDonald, 1976). Critically, the proportion of fusion responses (see “Fused” in Fig. 3) was smaller for Dynamic compared to Static and smaller for Eyes closed compared to Eyes open.

We tested whether these observations were statistically significant using a 2 (Motion: Static/Dynamic) × 2 (Eyes: Open/Closed) ANOVA with proportion of fusion responses as dependent variable. We found a significant main effect of Motion (*F*(1,138) = 7.98, *p* = 0.007, η_G_^2^ = 0.053) and a significant main effect of Eyes (*F*(1,138) = 5.07, *p* = 0.026, η_G_^2^ = 0.001), indicating that participants selected *fewer* fusion responses when motion preceded the speaker’s utterance and when the speaker’s eyes were closed (vs. open). The interaction effect was not significant (*F*(1,138) = 1.04, *p* = 0.308, η_G_^2^ < 0.001). Nonetheless, to assess whether the main effect of Eyes was present in both Motion conditions, we ran two pairwise *t*-tests comparing Eyes open to Eyes closed, separately for each Motion condition. The effect was significant in the Static condition (*t*(69) = 2.76, *p* = 0.007, Cohen’s *d* = 0.33) yet it was not significant in the Dynamic condition (*t*(69) = 0.76, *p* = 0.447, Cohen’s *d* = 0.09).

#### Response times

**Fig. 4:**
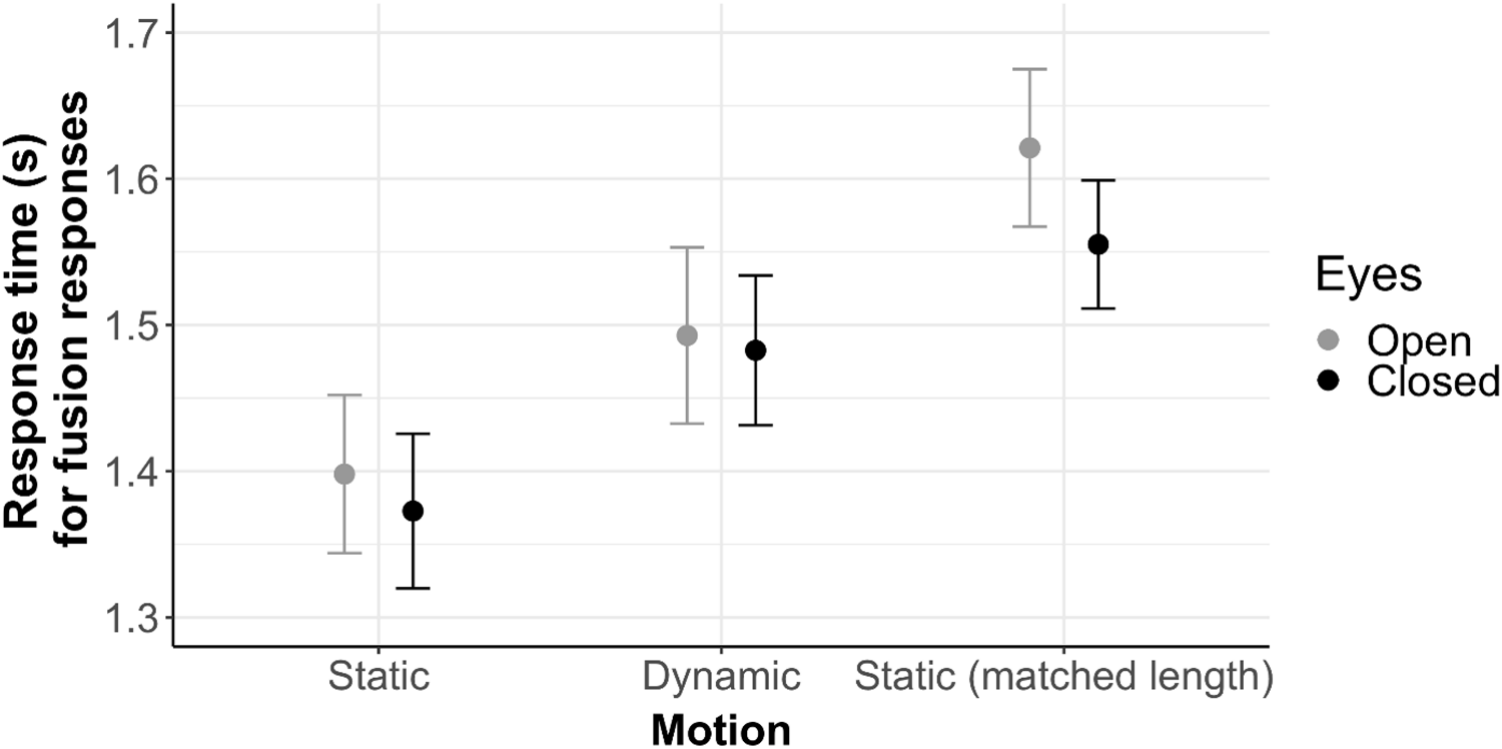
Response times in seconds are shown separately for Eyes open (grey) and Eyes closed (black), as a function of Motion (Static, Dynamic, Static (matched length)). Note that only fusion responses (i.e., when participants selected the fused syllable) are included. The Static condition with matched length, which was run as an additional control condition, is discussed in Sect. Control condition: Static matched

We determined whether these differences in participants’ response selection were reflected in their response times. Repeating the same 2 × 2 ANOVA with response times as dependent variable, we found no significant effects (Motion: *F*(1,138) = 1.82, *p* = 0.177, η_G_^2^ = 0.013; Eyes: *F*(1,138) = 1.42, *p* = 0.230, η_G_^2^ < 0.001; Motion x Eyes: *F*(1,138) = 0.26, *p* = 0.613, η_G_^2^ < 0.001); see Fig. 4.

#### Auditory and visual syllables

As the analysis of the fusion responses had shown that participants selected fewer fusion responses when eye motion preceded the speaker’s utterance and when the speaker’s eyes were closed, we aimed to find out which response (i.e., the auditory or the visual syllable) participants chose instead of the fusion response. To this end, we ran two further 2 × 2 ANOVAs, using the proportion of auditory syllables and the proportion of visual syllables as a dependent variable, respectively. These proportions indicate how often participants selected the auditory/visual syllable relative to the total number of responses.

For the proportion of auditory syllables, we found the mirror-inverted pattern of results as reported above for the proportion of fusion responses: a significant main effect of Motion (*F*(1,138) = 7.57, *p* = 0.008, η_G_^2^ = 0.051) and a trend towards significance for Eyes (*F*(1,138) = 3.37, *p* = 0.068, η_G_^2^ = 0.001). Again, the interaction effect was not significant (*F*(1,138) = 1.16, *p* = 0.275, η_G_^2^ < 0.001). For the proportion of visual syllables, we found no significant effects (Motion: *F*(1,138) = 0.01, *p* = 0.927, η_G_^2^ < 0.001; Eyes: *F*(1,138) = 0.72, *p* = 0.401, η_G_^2^ = 0.001; Motion x Eyes: *F*(1,138) = 0.002, *p* = 0.969, η_G_^2^ < 0.001). Taken together, these results indicate that participants selected the auditory syllable (what the speaker actually said) *more often* when eye motion preceded the speaker’s utterance and when the speaker’s eyes were closed (vs. open). Thus, under these conditions, participants selected the *auditory* syllable instead of the fusion response. This finding is in line with previous studies on the McGurk illusion which also showed that in incongruent trials where participants do *not* choose the fusion response, they typically choose the auditory rather than the visual syllable (e.g., Stropahl et al., 2017).

### Control condition: static matched

#### Rationale

A shortcoming of the present study is the fact that the length of the videos that were shown in the two between-subject conditions were of different length. Participants in the Static condition saw videos lasting 2 s whereas participants in the Dynamic condition saw videos lasting 3.5 s. Hence, it is possible that the difference in behavior observed between the two conditions is not a result of our manipulation (motion absent vs. present) but rather an effect of the video length. To deal with this potential confound, we ran an additional control condition in which we replicated the Static condition, yet matched it in length to the Dynamic condition. This was done by showing a still frame of the speaker for the first 1.5 s of the video—instead of the motion onset that was shown in the Dynamic condition.

#### Methods

A sample of 70 participants (32 females, 37 males, 1 other; *M* = 26.13 years, *SD* = 7.37 years) took part in the control condition. Design, procedure, and data analysis were the same as in the main experiment.

#### Results

Participants were highly accurate (*M* = 83%) in auditory-only trials and almost reached ceiling performance (*M* = 96%) in congruent trials (see Fig. 2). To test whether accuracy levels differed statistically in the auditory-only trials and congruent trials, we performed a one-way ANOVA with the within-subjects factor Eyes (Auditory only, Eyes closed (congruent), Eyes open (congruent)). As before, there was a main effect of Eyes (*F*(2,138) = 74.86, *p* < 0.001, *η*_*G*_^*2*^ = 0.343), showing that participants had a significantly lower accuracy in the auditory-only trials compared to the other two trial types (both corrected *p*s < 0.001; averaged Cohen’s *d* = 1.13). These differences in participants’ accuracy levels were also reflected in their response times (*F*(2,138) = 3.66, *p* = 0.030, *η*_*G*_^*2*^ = 0.011), with slower responses in the auditory-only trials.

As before, participants chose the fusion response in a high proportion of trials (*M* = 55%), indicating that they experienced the McGurk illusion (see Fig. 3). The proportion of fusion responses was descriptively, yet not significantly, smaller for Eyes closed compared to Eyes open, as shown by a paired *t*-test (*t*(69) = 1.57, *p* = 0.122, Cohen’s *d* = 0.19). Again, this difference was reflected in a mirror-inverted pattern for the auditory syllables, which were selected significantly more often in the Eyes closed compared to the Eyes open condition, as shown by a paired *t*-test (*t*(69) = 2.10, *p* = 0.039, Cohen’s *d* = 0.23).

When integrating the latter result into the overall context, it seems that the effect of Eyes (i.e., a reduced McGurk illusion when the speaker’s eyes are closed) is significant in the Static condition only, yet fails to reach significance in the Static matched condition (see above) and Dynamic condition (see Sect. Incongruent trials - Response choices). However, when looking at the data more closely, it turns out that the effect of Eyes depends on the basic size of the McGurk illusion (i.e., the percentage of perceived fused responses). In particular, the reason for the absence of the effect of Eyes in the Static matched and Dynamic conditions might be the generally smaller McGurk illusion in these conditions compared to the Static condition (Static matched: 55%; Dynamic: 62%; Static: 74%; see Fig. 3).

As we noticed that our data for the Static matched and Dynamic conditions seemed to be bimodally distributed, we considered performing a Median split to gain a better understanding of participants' behavior. To this end, we first assessed the degree of bimodality by calculating a bimodality coefficient (Pfister et al., 2013). In line with Pfister and colleagues, we considered a coefficient larger than 0.55 as an indication for bimodality. For both conditions, the computed coefficients surpassed this reference value (Static matched: 0.58; Dynamic: 0.59), suggesting that a Median split is a reasonable approach.

We first conducted a Median split for the Static matched condition and analyzed the above-Median and below-Median data sets separately. The results showed that for the above-Median data set, the size of the McGurk illusion is 82% when the speaker’s eyes are open and 78% when the speaker’s eyes are closed, resulting in a significant difference (*t*(34) = 3.16, *p* = 0.003, Cohen’s *d* = 0.31). Note that this effect size is comparable to the effect size of the Static condition. In contrast, for the below-Median data set, the size of the McGurk illusion is 29% when the speaker’s eyes are open and 30% when the speaker’s eyes are closed, showing no significant difference (*t*(34) = − 0.61, *p* = 0.546, Cohen’s *d* = − 0.05). When conducting the same Median split for the Dynamic condition, we find the same pattern for eyes open vs. closed (above-Median: 85% vs. 82%; below-Median: 40% vs. 41%). The difference between eyes open vs. closed for the above-Median data set is close to significant (*t*(34) = 1.86, *p* = 0.069, Cohen’s *d* = 0.29), yet it is not significant for the below-Median data set (*t*(34) =  − 0.58, *p* = 0.564, Cohen’s *d* = − 0.05).

To sum up, the effect of Eyes can be detected only if participants *reliably* perceive the McGurk illusion. Thus, the effect of Eyes can only be seen in those participants showing a large McGurk illusion (i.e., in the above-Median data set) but not in those showing a small McGurk illusion (i.e., in the below-Median data set).

Critically, we also compared the proportion of fusion responses in the control condition (Static matched) with the Static and the Dynamic conditions from the main experiment by conducting a 2 × 3 ANOVA with the within-subjects factor Eyes (Open, Closed), the between-subjects factor Motion (Static, Dynamic, Static matched), and with the proportion of fusion responses as a dependent variable. We found a significant main effect of Eyes (*F*(1,207) = 7.51, *p* = 0.006, η_G_^2^ = 0.001), indicating that participants selected fewer fusion responses when the speaker’s eyes were closed (vs. open). There was also a significant main effect of Motion (*F*(2,207) = 9.08, *p* < 0.001, η_G_^2^ = 0.079). The interaction effect was not significant (*F*(2,207) = 0.54, *p* = 0.542, η_G_^2^ < 0.001). We followed up the main effect of Motion with pairwise comparisons. There was a significant difference between Static and Static matched (*t*(138) = 4.22, *p* < 0.001, Cohen’s *d* = 0.71), with a higher proportion of fusion responses in Static. There was no significant difference between Dynamic and Static matched (*t*(138) = 1.49, *p* = 0.138, Cohen’s *d* = 0.25). On a descriptive level, however, there was a higher proportion of fusion responses in Dynamic. This result indicates that the extent to which participants in our control condition—which was identical to the Static condition yet matched in length to the Dynamic condition—experienced the McGurk illusion was more similar to the Dynamic condition than to the Static condition (see Fig. 3).

We performed the same 2 × 3 ANOVA with response times as the dependent variable. The pattern of results mirrored the analysis for fusion responses (see Fig. 4). There was a significant main effect of Eyes (*F*(1,207) = 5.35, *p* = 0.022, η_G_^2^ = 0.001), indicating that participants were faster to select a response when the speaker’s eyes were closed (vs. open). This suggests that when the speaker’s eyes were closed, participants were more likely to choose the accurate auditory response (instead of the fusion response) and to make this response faster compared to when the speaker’s eyes were open﻿. There was also a significant main effect of Motion (*F*(2,207) = 3.90 *p* = 0.022, η_G_^2^ = 0.034). The interaction effect was not significant (*F*(2,207) = 1.30, *p* = 0.275, η_G_^2^ < 0.001). We followed up the main effect of Motion with pairwise comparisons. There was a significant difference between Static and Static matched (*t*(138) = 2.90, *p* = 0.004, Cohen’s *d* = 0.49), with slower responses in Static matched. There was no significant difference between Dynamic and Static matched (*t*(138) = 1.40, *p* = 0.164, Cohen’s *d* = 0.24). On a descriptive level, however, responses were slower in Static matched. This result indicates that participants were fastest to respond in the Static condition, distinctively slower in the Dynamic condition, and again slightly slower in the Static matched control condition (see Fig. 4).

## Discussion

In the present study, we investigated whether a speaker’s gaze behavior (i.e., motion and eye contact) dynamically captures an addressee’s attention and thereby influences how the addressee processes the speaker’s audiovisual speech signals.

To this end, we used the classic McGurk illusion and manipulated whether the speaker (a) moved his eyelids up/down (i.e., opened/closed his eyes) prior to speaking or did not show any eye motion, and (b) spoke with open or closed eyes. When the speaker’s eyes moved (i.e., opened or closed) before an utterance, and when the speaker spoke with closed eyes, the McGurk illusion was weakened (i.e., addressees reported significantly fewer illusory percepts). It seems that these two main effects can be traced back to two separate mechanisms, as outlined below.

### Effect of motion

When looking at the *main experiment*, the effect of motion seems to highlight the power of motion cues to capture people’s attention, converging with previous research on the effects of sudden onset (eye) motion on attentional capture (e.g., Abrams & Christ, 2003; Böckler et al., 2014, 2015; van der Wel et al., 2018). In particular, the sudden opening/closing of the speaker’s eyes presumably directed participants’ attention to the eyes (and away from the lips). Thus, the misleading visual signal provided by the speaker’s lips had a smaller influence on the audiovisual integration process such that participants perceived fewer illusory percepts and instead perceived the actual auditory syllables. This finding is in line with previous research showing that the degree to which people perceive the McGurk illusion depends on their attentional focus on the speaker’s mouth vs. elsewhere, with a focus on the mouth leading to an increase of the illusion (Gurler et al., 2015; Stacey et al., 2020). It has also been shown that people only experience the McGurk illusion if they consciously attend to the speaker’s lips (Munhall et al., 2009). Thus, the effect of motion may be interpreted in terms of attentional capture.

However, when considering the *control experiment* (“Static matched”), this interpretation is called into question, as the results show that a static stimulus that matched the motion stimulus in length also led to a reduced McGurk illusion. From this result, one might conclude that the motion itself did not actually play the main role, but it was rather the video length that caused the difference between the Static (motion absent, shorter video) and the Dynamic condition (motion present, longer video) in the main experiment. Some additional differences between the Static matched and Dynamic conditions should be considered, however. Specifically, participants in the Static matched condition (1) experienced the McGurk illusion to an even smaller extent than participants in the Dynamic condition and (2) responded more slowly than in the Dynamic condition. These two aspects in combination suggest that different processes might be at work in the two conditions, yet leading to similar outcomes. In particular, it is possible that the reduced McGurk illusion in the Dynamic condition is caused, at least partially, by the motion cue functioning as attentional capture (as discussed above). In the Static matched condition, however, the reduced McGurk illusion might be caused by the fact that participants were generally less attentive because the still frame at the beginning of each video is rather boring. This general reduction of attention might lead to (1) a reduction in audiovisual integration (cf. Alsius et al., 2005; Munhall et al., 2009; Talsma & Woldorff, 2005) and (2) a slow-down of responses; just as observed in the Static matched condition.

### Effect of eye contact

Our results suggest that the McGurk illusion is smaller when the eyes of the speaker were closed compared to open–provided that people reliably perceive the basic McGurk illusion (regarding the latter constraint, please see Sect. Control condition: Static matched - Results). First of all, it is possible that when the speaker spoke with closed eyes, participants did not consider themselves the intended recipient of the message and thus paid less attention overall to the stimuli. Since sufficient attention is a prerequisite for audiovisual integration to occur (Alsius et al., 2005; Munhall et al., 2009; Talsma et al., 2007), a general reduction of attention might have impaired the integration process, thus resulting in fewer illusory percepts when the speaker’s eyes were closed.

The effect of eye contact might also suggest that a speaker’s closed eyes capture the addressee’s attention more strongly than open eyes in the setting of the present study. One reason could be that closed eyes in a conversation are very unusual and thus salient for an addressee. Typically, interlocutors converse with open eyes following specific gaze patterns (Ho et al., 2015), yet the speaker in the present study spoke with closed eyes. Presumably, the fact that speaking with closed eyes is not consistent with common social norms led to increased salience and thus caught and captured participants’ attention. Apart from the fact that closed eyes might be more salient than open eyes in a conversation context, it is also possible that participants focused more on the speaker’s closed compared to his open eyes because feeling another’s direct gaze elicits self-referential processing (“Watching Eyes model”, see Conty et al., 2016; Hietanen & Hietanen, 2017) and self-awareness (e.g., Baltazar et al., 2014; Hazem et al., 2017; Pönkänen et al., 2011), increases arousal (Helminen et al., 2011), and invites for social interaction (Ho et al., 2015). Thus, in line with the finding that people avoid long eye contact with strangers (Ellsworth et al., 1972; Laidlaw et al., 2011) and look longer at faces with averted than direct gaze (Helminen et al., 2011), participants in the present study might have preferred to attend to the speaker’s eyes when those were closed and they were not feeling watched, as this creates less self-involvement. Thus, the effect of eye contact can be interpreted in terms of the saliency of closed eyes in a conversation context as well as in terms of the Watching Eyes model (cf. Conty et al., 2016), as both mechanisms would result in fewer illusory percepts when the speaker’s eyes were closed.

A future lab-based study could use eye tracking to measure gaze fixations as a proxy of overt attention and pupil size as a proxy of attentional processing to disentangle the two interpretations. Specifically, the interpretation that a speaker’s closed eyes reduced the illusion due to generally reduced attention would be supported by gaze patterns that reflect disengagement of the visual scene (e.g., fewer fixations on the face) in the Eyes closed condition. By contrast, the interpretation that the reduced illusion is due to attention capture by closed eyes would be supported by earlier, more and/or longer fixations at the eye region in the Eyes closed condition. Finally, it is also possible that both interpretations are partially correct, as they are not mutually exclusive.

### Study limitations

Some of our findings warrant additional research before stronger conclusions can be drawn. Regarding our interpretation of the motion cue in terms of attentional capture, it is possible that apart from the eyes, other motion cues in the speaker’s face (e.g., wrinkling the forehead) might have similar effects. It has been shown, for instance, that an external moving object (i.e., a falling leaf in front of a speaker’s face) reduces the McGurk illusion (Tiippana et al., 2004). It is noteworthy that in this study, the leaf motion occurred *during* the speaker’s utterance whereas the motion in the present study occurred *prior* to the utterance. Future studies are needed to determine the specificity and boundary conditions of the motion effect, especially regarding the ‘identity’ and the timing of the motion.

More generally, regarding the reduced McGurk illusion in the Dynamic condition (motion present, longer video) compared to the Static condition (motion absent, shorter video), we cannot argue conclusively as to whether it was the motion or the length of the video that caused this effect. The results of the control condition (“Static matched”: motion absent, longer video) resembled those of the Dynamic condition, suggesting, at first glance, that the effect can be attributed to the video length. However, as pointed out above, it is possible that different processes were at work in the Dynamic condition (motion cue guides addressee’s attention to the speaker’s eyes) and the Static matched condition (still frame leads to addressee feeling bored and thus paying less attention overall). Both processes would result in an impaired integration and thus in fewer illusory percepts. Further control experiments with systematic and orthogonal manipulations of motion and video length are needed to resolve this issue.

The effect of eye contact (i.e., a smaller McGurk effect when the speaker’s eyes are closed) in the present study is rather small and not significant in all pairwise comparisons. However, the effect is significant when analysed with an ANOVA with higher statistical power (see Sect. Control condition: Static matched - Results) and the effect (or a tendency, at least) occurs in all of the three conditions, indicating that it is reliable. Future research could identify potential factors that might increase the size of the effect or abolish it altogether.

When it comes to the generalizability and applicability of findings from the McGurk illusion, one should bear in mind that the processing of the McGurk illusion does not necessarily generalize to natural forms of audiovisual speech processing (for reviews, see Alsius et al., 2018; Rosenblum, 2019) as, for instance, audiovisual sentence recognition abilities do not predict one’s susceptibility to the McGurk illusion (van Engen et al., 2017) and distinct brain regions are active during the perception of audiovisual speech and the McGurk illusion (Erickson et al., 2014). Further systematic studies are thus needed to investigate in how far findings from the McGurk illusion extend to audiovisual speech perception more generally.

### Conclusion

The present findings contribute to an ongoing debate on whether multisensory integration is an automatic process or whether it can be affected by attentional processes (for reviews, see Talsma et al., 2010; Ten Oever et al., 2016; Spence & Frings, 2020). In line with earlier work (Alsius et al., 2005; Tiippana et al., 2004), our findings support the view that multisensory integration is susceptible to attentional manipulations in the case of audiovisual speech processing. In particular, our findings suggest that attentional focus on the speaker’s eyes affects the integration process in the McGurk illusion. Future studies could expand this work by investigating if attention-capturing stimuli in the auditory domain (e.g., a word spoken prior to the McGurk syllable) also affects the integration process or if the observed effects are specific to the visual modality.

In sum, the present study shows that speaker gaze affects audiovisual integration in the McGurk illusion. Our results thereby demonstrate that a speaker’s gaze behavior can dynamically capture an addressee’s attention, influencing how the addressee processes the speaker’s audiovisual speech signals. These findings reaffirm the power of speaker gaze to guide attention, showing that its dynamics can modulate low-level processes such as the integration of multisensory speech signals.

## Data Availability

All data and stimuli are publicly available via the Open Science Framework.

## Footnotes

1 Based on previous studies showing that the McGurk illusion can be reliably found in online studies, we were confident that conducting our study online should not be problematic, as we should be able to replicate the McGurk illusion.

2 Due to the fact that participants were recruited online, we could only collect basic demographic information. As a result, we do not know for sure whether English was participants’ native language or whether they learned English as a second (or third) language. We do know, however, that between 20 and 30% of our participants originated from the US or UK and hence should be native English speakers; most of the other remaining 70–80% originated from European countries where English is not the first language. (In this respect, the samples in our three conditions did not differ noticeably.) This aspect could be relevant to the present study because phonetic perception is influenced by one’s native language. We thus cannot exclude the possibility that differences in our participants’ first language might have affected the extent to which they perceived the McGurk illusion. Moreover, we could not collect information regarding participants’ sociocultural background and can thus not exclude the possibility that differences in our participants’ sociocultural norms might have affected how they interpret eye contact in communicative contexts. We acknowledge these shortcomings in our method, yet believe that our rather large sample size should have helped to average out potential inter-individual differences.

3 It is important to consider that temporal precision in *online* studies is not as good as in *lab-based* studies. However, a recent study comparing the performance of different popular packages for online studies (Bridges et al., 2020) showed that PsychoPy (which we used for the present study) achieves a precision under 3.5 ms for response times in all browsers, making it the most precise among all packages tested. We consider this level of precision sufficient for the present study in which response times lie in a range of 1.4–1.6 s and differences in response times between conditions are around 100 ms.

## Code availability

The code is freely available upon request.
